# Case Report: Parachute tricuspid valve in association with cyanotic congenital heart disease and juxtaposed atrial appendages in a 1-year-old boy—a rare anatomical association

**DOI:** 10.3389/fcvm.2026.1683429

**Published:** 2026-02-19

**Authors:** Devender Sangwan, Ashutosh Marwah, Vaishali Jain

**Affiliations:** 1 Pediatric Cardiology, Fortis Hospital, Delhi, India; 2 Pediatric Cardiology, Fortis Escorts Heart Institute and Research Centre, New Delhi, India

**Keywords:** cyanotic congenital heart disease, double outlet right ventricle, parachute tricuspid valve, pulmonary stenosis, ventricular septal defect

## Abstract

Parachute tricuspid valve is an exceedingly rare congenital anomaly, often described in association with acyanotic heart defects. Its occurrence alongside cyanotic congenital heart disease is even less common. We report the case of a 1-year-old boy presenting with complex cyanotic heart disease along with parachute tricuspid valve. Detailed evaluation revealed a constellation of anomalies, including parachute tricuspid valve, double outlet right ventricle with D-malposed great arteries, severe pulmonary stenosis, and a non-routable ventricular septal defect. Echocardiography played a vital role in defining the complex anatomy and guided the decision toward single-ventricle palliation. This case underscores the importance of meticulous anatomical assessment in patients with congenital heart disease and contributes to the sparse literature on this rare condition.

## Introduction

Double outlet right ventricle (DORV) is a congenital cardiac malformation in which both great arteries arise predominantly from the right ventricle. Systemic output in DORV depends on the size and routability of the ventricular septal defect. Juxtaposed atrial appendages refer to an abnormal spatial arrangement in which both atrial appendages lie on the same side of the great arteries. Parachute valve anomalies are characterized by insertion of all chordae tendineae into a single papillary muscle (1). While the parachute mitral valve is a well-recognized component of Shone's complex and other left-sided obstructive lesions, the tricuspid variant is rarely reported.

We report a rare case of parachute tricuspid valve associated with complex cyanotic congenital heart disease in a 1-year-old child. The associated anomalies included double outlet right ventricle with D-malposed great arteries; a large, non-routable inlet ventricular septal defect; severe infundibular and valvar pulmonary stenosis; fossa ovalis atrial septal defect; juxtaposed atrial appendages; and a right-sided aortic arch. The rarity of parachute tricuspid valve in association with such complex cyanotic heart disease makes this case noteworthy.

## Case report

A 1-year-old male child was referred for evaluation of persistent cyanosis and failure to gain weight since early infancy. There was no antenatal diagnosis, and fetal echocardiography had not been performed. There was no family history of congenital heart disease or consanguinity. Cyanosis was noted within the first few weeks of life and progressively worsened. The child had poor weight gain but no prior cardiac interventions.

At presentation, the child weighed 7.9 kg and measured 75 cm in height. Oxygen saturation on room air ranged from 72% to 76%. There was a grade 3/6 ejection systolic murmur best heard at the left upper sternal border.

**Figure 1:**
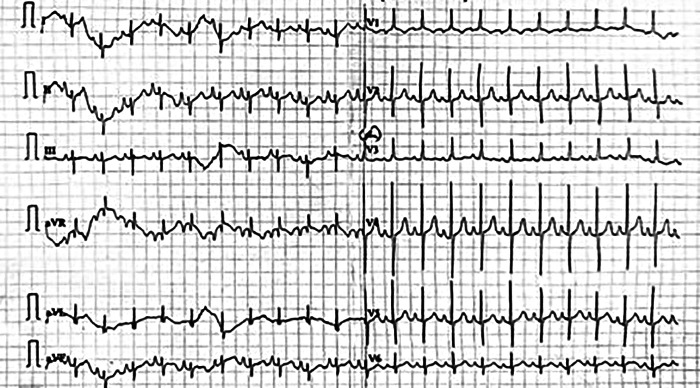
Full standardised ECG shows HR = 125/min, sinus rhythm with a normal QRS axis. There is evidence of right atrial enlargement.

Chest radiography revealed mild cardiomegaly with a narrow mediastinum and oligemic lung fields. Cardiac situs was normal, and the aortic arch was right-sided. Electrocardiography demonstrated sinus rhythm with a normal QRS axis, right atrial enlargement, and right ventricular hypertrophy (Figure 1).

Cross-sectional echocardiography and Doppler interrogation revealed situs solitus, levocardia, and concordant atrioventricular connections. Both systemic and pulmonary venous drainage were normal. A 6-mm fossa ovalis atrial septal defect with right-to-left shunt was present. The atrial appendages were juxtaposed to the left.

**Figure 2:**
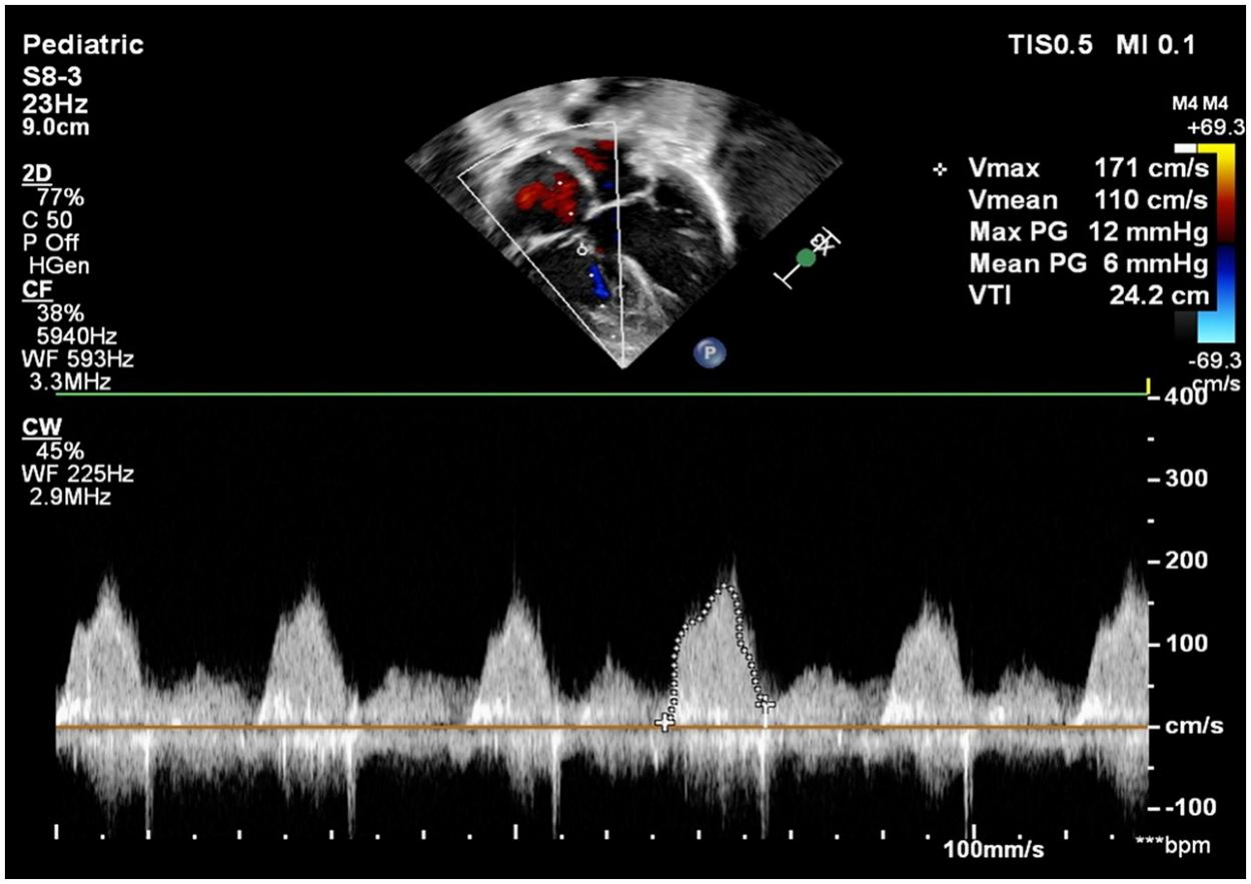
Continuous wave doppler at tricuspid inflow flow showing tricuspid valve stenosis mean gradient of 6 mmHg across tricuspid valve.

**Figure 3:**
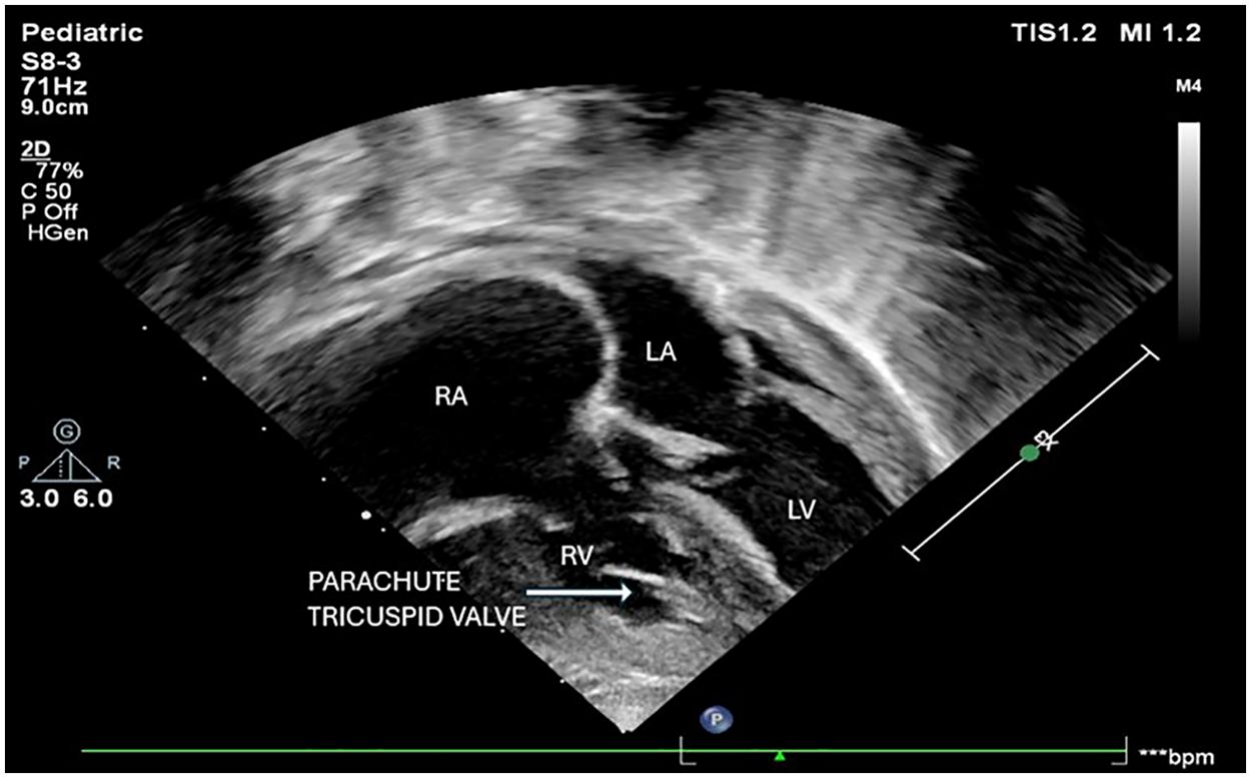
Apical four chamber view showing single papillary muscle on septal surface with chordal attachment from septal and anterior leaflets of tricuspid valve. Right atrium (RA), Right ventricle (RV), Left atrium (LA), Left ventricle (LV).

The tricuspid valve annulus measured 11 mm (Z score −2.3). Color and continuous-wave Doppler interrogation revealed flow acceleration across the tricuspid valve with a mean gradient of 6 mmHg (Figure 2). Further evaluation showed that all chordae of the tricuspid valve were inserted into a single papillary muscle attached near the septal surface of the right ventricle, consistent with parachute tricuspid valve (Figure 3). No tricuspid regurgitation was noted.

The morphological right ventricle was hypoplastic. Both great arteries originated from the right ventricle, with D-malposition of the great vessels. There was severe pulmonary stenosis with a peak gradient of 78 mmHg. The left ventricle was of normal size and communicated with the right ventricle through a non-restrictive, non-routable inlet ventricular septal defect. Branch pulmonary arteries were of acceptable size for age, and biventricular systolic function was preserved.

The combination of a non-routable ventricular septal defect, severe pulmonary stenosis, hypoplastic right ventricle, and tricuspid inflow obstruction precluded biventricular repair. The child underwent atrial septectomy along with a bidirectional Glenn shunt. No intervention was done on the tricuspid valve. Intraoperative findings confirmed the echocardiographic anatomy. The postoperative course was uneventful, and the patient was discharged on postoperative day 7. Postoperative echo on day 7 revealed good ventricular function, good interatrial communication, no gradient across the tricuspid valve, and a patent and well-functioning bidirectional Glenn shunt.

## Discussion

Parachute tricuspid valve is a rare abnormality, far less commonly reported than its mitral counterpart. The defining feature of this anomaly is the insertion of all tricuspid chordae into a single papillary muscle, resulting in varying degrees of tricuspid inflow obstruction. Most reported cases have been associated with acyanotic cardiac defects such as atrial or ventricular septal defects (2, 3). In the published literature, only two cases have been reported in association with complex cardiac defects—one with Tetralogy of Fallot (4) and another with congenitally corrected transposition of great arteries (5).

Recognition of tricuspid inflow obstruction is particularly important when biventricular repair is contemplated. In the presence of a right-to-left atrial shunt, it is often difficult to assess tricuspid valve stenosis, and one may underestimate or completely miss the stenosis on Doppler alone. However, a properly performed cross-sectional study can clearly document chordal attachments and detect the abnormality. In the presence of tricuspid stenosis, complete closure of the atrial communication can cause a rise in atrial pressure and lead to deleterious effects upon hepatic and splanchnic circulation. Leaving a small atrial communication or adding a superior cavopulmonary anastomosis may be needed in such patients.

In our patient, the presence of a large inlet ventricular defect, double outlet right ventricle, and D-malposed aorta precluded a biventricular option. The patient underwent a bidirectional Glenn shunt with enlargement of atrial communication. The patient remains under regular follow-up and is scheduled for Fontan completion after 2 years.

Echocardiography played a vital role in delineating the anatomical details and surgical planning.

Case reports of parachute tricuspid valve with other associated heart disease mentioned in Table 1 have been described previously by Yuan et al. (6).

**Table 1:** Published case reports of parachute tricuspid valve.

Year	Author	Age	Sex	Papillary muscle	Associated anomalies	TV dysfunction	Diagnostic modality	Treatment	Outcome
1979	Milo et al.	Neonate (op at 6 years 8 months)	M	Single	DORV, straddling MV, RVOT obstruction, inlet VSD	TS	Autopsy	MV repair + VSD closure	Died
1979	Ariza et al. (4)	Neonate	NR	Single	TOF with pulmonary atresia	TS	Echo	NR	NR
1980	Maitre Azcarate	NR	NR	Single (apical)	Cor triatriatum	Leaflet fusion	Autopsy	None	Died
1997	Godart et al.	Neonate (followed to 2 years)	F	Single anterior	CCTGA, VSDs, hypoplastic arch, CoA, PDA, PAH	Severe TS (annular ring)	Echocardiography	Annular ring resection + TVR	Died (septic shock)
2006	Marwah et al. (2)	21 years	F	Single	ASD, VSD	Mild TS/near-normal	TTE	Follow-up	Unchanged
2010	Uçar et al.	25 years	M	Single	VSD	TR	TTE	Follow-up	NR
2012	Demirkol et al.	21 years	M	Single	Isolated PTV	Trivial TR	TTE, 3D-TTE, 3D-TEE, CTA	Follow-up	Stable
2012	Demirkol et al.	24 years	M	Single (bifid)	ASD	Trivial TR	TTE, 3D-TEE, CTA	Surgical ASD closure	Recovered
2012	Mohan et al. (3)	30 years	NR	Single (short)	ASD, PAH	TR	TTE, Cardiac CT	Follow-up	NR
2013	Kurtul et al.	33 years	M	Single	PMV, VSD, PAH, anomalous LCx	Normal TV function	TTE	Transcatheter VSD closure	Recovered
2015	Mohan et al. (5)	23 years	F	Single	PMV, CCTGA, PS, BAV	Mild TR	TTE	Follow-up	Unchanged
2016	Gupta et al.	7.5 months	M	Single	ASD, VSD	Severe TS	TTE	VSD closure + chordal transfer + ASD closure	Recovered
2017	Alimi and Fazlinezhad	52 years	F	Single (calcified)	ASD	Moderate–severe TR	TTE, 3D-TEE	ASD device closure	Improved
2017	Alimi and Fazlinezhad	30 years	F	Single (calcified)	None	Mild–moderate TR	TTE, 3D-TTE	Follow-up	No change

[Data summarized from Yuan et al. (6)]. ASD, atrial septal defect; BAV, bicuspid aortic valve; CCTGA, congenitally corrected transposition of great arteries; CoA, coarctation of aorta; CTA, computed tomographic angiography; DORV, double outlet right ventricle; MV, mitral valve; PAH, pulmonary artery hypertension; PMV – parachute mitral valve; PS – pulmonary stenosis; PTV, parachute tricuspid valve; TR, tricuspid regurgitation; TS, tricuspid stenosis; TVR, tricuspid valve replacement; VSD, ventricular septal defect.

However, to the best of our knowledge, the occurrence of parachute tricuspid valve in association with double outlet right ventricle, D-malposed great arteries, severe pulmonary stenosis, non-routable ventricular septal defect, and juxtaposed atrial appendages has not been previously reported.

## Conclusion

This report describes an exceptionally rare constellation of congenital cardiac anomalies, including parachute tricuspid valve, double outlet right ventricle with D-malposed great arteries, severe pulmonary stenosis, non-routable ventricular septal defect, and juxtaposed atrial appendages. These findings resulted in severe cyanosis and necessitated a single-ventricle palliation strategy. The case highlights the importance of detailed echocardiographic assessment in complex congenital heart disease, using both two-dimensional and color Doppler to achieve a complete diagnosis. Furthermore, the report also demonstrates how a rare atrioventricular valve abnormality can affect surgical decision-making.

## Data Availability

The datasets presented in this study can be found in online repositories. The names of the repository/repositories and accession number(s) can be found in the article/Supplementary Material.

## References

[1] Swan H Trapnell JM Dens TJ. Congenital mitral stenosis and systemic right ventricle with associated pulmonary vascular changes frustrating surgical repair of patent ductus arteriosus and coarctation of the aorta. Am Heart J. (1949) 38(6):914–23. 10.1016/0002-8703(49)90892-3 15395923

[2] Marwah A Suresh PV Shah S Misra A Maheshwari S. Parachute tricuspid valve. Eur J Echocardiogr. (2006) 7:226–7. 10.1016/j.euje.2005.10.002 16290132

[3] Mohan JC Shekhar C Mohan V Kaur B Singh SK. Parachute tricuspid valve in an asymptomatic adult. Indian Heart J. (2012) 64(1):93–4. 10.1016/S0019-4832(12)60020-0 22572436 PMC3861073

[4] Ariza S Cintado C Castillo JA Descalzo A Cañadas M Santos J Parachute tricuspid valve associated with fallot’s tetralogy. Arch Mal Coeur Vaiss. (1979) 72(3):317–20. 114142

[5] Mohan JC Shukla M Sethia A. Parachute deformity of both atrioventricular valves with congenitally corrected transposition in an adult. Indian Heart J. (2015) 67(6):565–9. 10.1016/j.ihj.2015.06.012 26702687 PMC4699942

[6] Yuan SM. Parachute tricuspid valve: a systematic review. Orphanet J Rare Dis. (2020) 15(1):305. 10.1186/s13023-020-01561-y 33115523 PMC7592550

